# Treatment pathways and disease journeys differ before and after introduction of novel agents in newly diagnosed multiple myeloma in Taiwan

**DOI:** 10.1038/s41598-020-80607-4

**Published:** 2021-01-13

**Authors:** Yanfang Liu, Chao-Hsiun Tang, Hong Qiu, Sarah Siggins, Hsin-An Hou

**Affiliations:** 1 Global Epidemiology, Janssen Research and Development, Singapore, Singapore; 2 School of Health Care Administration, College of Management, Taipei Medical University, Taipei, Taiwan; 3 Global Epidemiology, Janssen Research and Development, Titusville, USA; 4 Janssen Medical Affairs Asia Pacific, Sydney, Australia; 5 Division of Hematology, Department of Internal Medicine, National Taiwan University Hospital, 7 Chung Shan S. Rd., Taipei, 10002 Taiwan

**Keywords:** Cancer epidemiology, Haematological cancer, Haematological diseases, Cancer, Diseases, Medical research, Epidemiology, Outcomes research

## Abstract

Limited real-world data are available regarding treatment practices and outcomes of multiple myeloma (MM) in Asia. We conducted a retrospective cohort study using the Taiwan National Healthcare Insurance Research database and Taiwan Death Registry and used a Markov model to describe disease progression and outcomes in 4092 patients newly diagnosed with MM from 01-Jan-2007 to 31-Dec-2015. We observed marked differences in the characteristics, length and outcome of the clinical journey between patients who did/did not receive autologous stem cell transplant, and between patients initiated on novel agents versus other treatment regimens. In transplant recipients, initiation with combined thalidomide + bortezomib increased over time (12.2–77.5%). Progression-free survival after first-line treatment improved and a lower percentage of patients died. Lenalidomide in second and third-line regimens increased (15.5–31.5%). In non-transplanted patients, initiation with novel agents increased (17.5–54.6%), but death rates remained high (60.3%). The treatment landscape of MM in Taiwan has evolved, with increased use of combined bortezomib + thalidomide for first-line and lenalidomide for second/third-line but many patients die before receiving second-line treatment. Novel agents with different modes of action should be used as early as possible to maximize their benefits. Improved MM treatments remains a critical medical need.

## Introduction

Multiple myeloma (MM) remains incurable. However, greater understanding of its underlying cellular and molecular biology has driven the development of novel therapeutics. Survival rates have improved by as much as 50% since the introduction of targeted proteasome inhibitors such as bortezomib, and immunomodulatory drugs including thalidomide and lenalidomide, combined with increasing use of high-dose therapy prior to autologous stem cell transplantation (ASCT)^1–5^. The incidence of MM in Taiwan is increasing at one of the highest rates in the world, along with China and Korea^6^. In a previous study, we used the National Health Insurance Research Database (NHIRD), a database holding all administrative and claims data related to healthcare in the entire Taiwanese population, to describe the epidemiology of MM in Taiwan^7^. We showed that crude MM incidence increased by 40% between 2007 and 2015 in Taiwan and that prevalent MM cases increased by more than 60%^7^. A decrease in case fatality over the same period (from 25.5% in 2007 to 18.3% in 2015) was co-incident with the re-imbursement of novel agents for the first-line treatment of MM in Taiwan: thalidomide in July 2009, bortezomib in June 2012, and lenalidomide for patients with first-line treatment failure in December 2012. ASCT is reimbursed in Taiwan for patients with MM who are transplant eligible.

Several analyses describing MM treatment in clinical practice have been published in recent years; most describe single or multi-center studies^8–11^, and real-world data on MM treatment patterns are not available in Asian countries. We used the NHIRD to describe treatment pathways for MM and linked this information to the Taiwan Death Registry, using progression through lines of treatment and mortality as indicators of disease progression after diagnosis. Disease progression models (DPMs) were developed to understand how patients moved from first, to second, and third-line therapy, ASCT, and death in the periods before and after widespread bortezomib use in Taiwan. This is the first study to comprehensively investigate MM treatment patterns in Asia using populaton-based databases. The congruent availability of historical and contemporary data provide unique insights into temporal trends, and can be used in health technology assessment evaluations, and for developing treatment guidelines and reimbursement policy.

## Results

Two exposure periods were defined according to the bortezomib recommendation date of June 2012: the pre-bortezomib period included patients diagnosed from 01 January 2007 until 31 May 2012, and the post-bortezomib period from 01 June 2012 until 31 December 2015.

**Figure 1:**
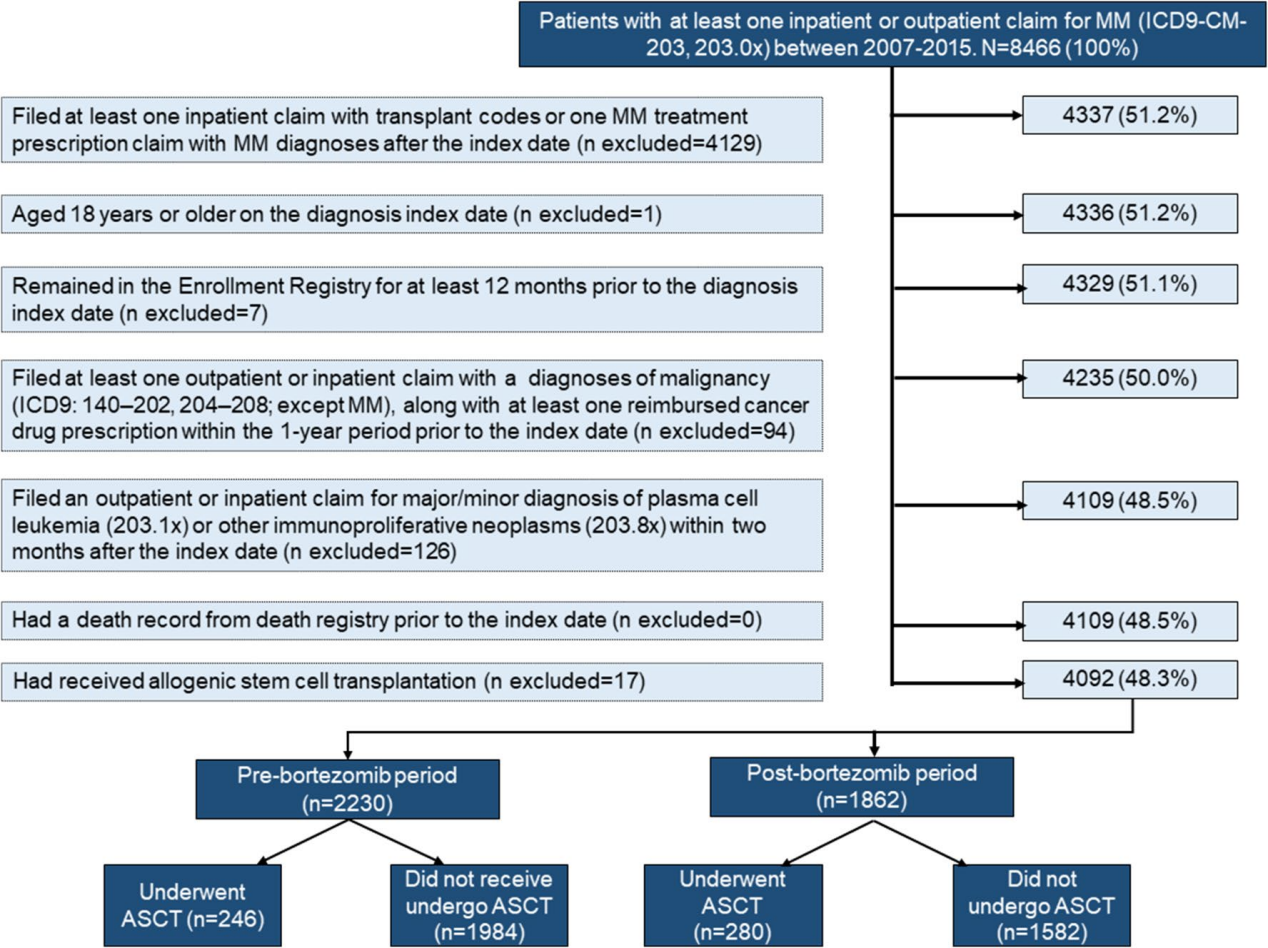
Patient selection flow-chart. *ASCT* autologous stem cell transplantation, *IQR* inter-quartile range, *MM* multiple myeloma.

There were 4092 patients included in the cohort analysis of treatment; 2230 in the pre-bortezomib period of whom 246 underwent ASCT (11.0%), and 1862 in the post-bortezomib period of whom 280 underwent ASCT (15.0%) (Fig. 1). The mean follow-up period was 5.69 years (standard deviation [SD] 2.4) in ASCT recipients in the pre-bortezomib period, and 3.05 (SD 2.8) years in non-transplanted patients. In the post-bortezomib period the mean follow-up period was 3.26 (SD 1.2) and 2.21 (SD 1.5) years, respectively.

### Patient characteristics in the pre-bortezomib period

Demographic and clinical characteristics of patients diagnosed in the pre-bortezomib period are provided in Table S1. Briefly, among 246 ASCT recipients in the pre-bortezomib period, 58.5% were male and the mean age was 53.9 years (SD 7.2) at the time of MM diagnosis. The mean interval between diagnosis and transplantation was 1.04 (SD 1.06) years. The vast majority (96%, 236/246) of ASCT recipients were < 65 years of age. Of 1984 non-transplanted patients, 57.3% were male and the mean age was 69.4 years (SD 11.6).

Approximately 41% of ASCT recipients diagnosed in the pre-bortezomib period received first-line treatment with novel agents alone (NA), 19% received chemotherapy combined with novel agents (CCNA), 24% received chemotherapy alone (CA) and 16% received steroids alone (SA).

First-line treatment in non-transplanted patients was most often with CA (30%), CCNA (28%), or SA (24%), and 18% received NA.

### Patient characteristics in the post-bortezomib period

Compared to the pre-bortezomib period, the gender balance of ASCT recipients in the post-bortezomib period (N = 289) had equalized (52.5% were male) and the mean age increased to 57.0 years (SD 7.7) at the time of MM diagnosis (Table 1) with mean interval between MM diagnosis and transplantation shortened to 0.8 (SD 0.6) years. The age-distribution of ASCT recipients was similar in both periods, although the number of patients aged ≥ 65 years who received ASCT increased from 10 (0.7%) in the pre-bortezomib period to 40 (3.6%) in the post-bortezomib period. As observed in the pre-bortezomib period, 1582 non-transplanted patients in the post-bortezomib period were older than ASCT recipients (69.6 vs 57.0 years), and a higher proportion were aged ≥ 65 years (67.3% vs 14.3%). More non-transplanted patients had co-morbidities at diagnosis than ASCT recipients, and the CCI was 1.5 versus 0.7, respectively.

**Table 1:** Demographic, clinical characteristics and follow-up duration of patients with treated MM diagnosed in the post-bortezomib period (1 June 2012 until 31 Dec 2015) by ASCT status and first-line treatment.

Variables	Patients who underwent ASCT (n = 280)		Patients without ASCT (n = 1582)			
	NA	CCNA	NA	CCNA	CA	SA
	N = 213	N = 63	N = 863	N = 466	N = 44	N = 209
**Sex n (%)**						
Male	114 (53.5)	30 (47.6)	461 (53.4)	260 (55.8)	18 (40.9)	104 (49.8)
Female	99 (46.5)	33 (52.4)	402 (46.6)	206 (44.2)	26 (59.1)	105 (50.2)
**Age (mean, SD)**	56.9 (7.9)	57.7 (6.6)	68.3 (11.6)	70.7 (10.2)	74.1 (12.4)	71.6 (12.7)
18–29	**	0 (0.0)	**	0 (0.0)	0 (0.0)	**
30–39	8 (3.8)	0 (0.0)	6 (0.7)	**	0 (0.0)	**
40–49	26 (12.2)	6 (9.5)	40 (4.6)	19 (4.1)	**	11 (5.3)
50–59	88 (41.3)	28 (44.4)	154 (17.8)	39 (8.4)	5 (11.4)	22 (10.5)
60–69	85 (39.9)	25 (39.7)	251 (29.1)	126 (27.0)	10 (22.7)	39 (18.7)
70–79	5 (2.3)	4 (6.3)	246 (28.5)	190 (40.8)	10 (22.7)	70 (33.5)
≥ 80	0 (0.0)	0 (0.0)	165 (19.1)	90 (19.3)	18 (40.9)	64 (30.6)
**Follow up duration**^**a**^** (years)**						
Mean (SD)	3.25 (1.2)	3.28 (1.2)	2.21 (1.5)	2.26 (1.5)	2.22 (1.6)	2.10 (1.6)
Med (range)	3.1 (0.56–5.55)	3.2 (0.57–5.46)	2.14 (0.03–5.55)	2.27 (0.05–5.56)	2.12 (0.08–5.07)	2.21 (0.02–5.55)
Q1, Q3	2.36, 4.16	2.43, 4.12	0.82, 3.28	0.78, 3.34	0.72, 3.95	0.42, 3.37
**Comorbidity n (%)**						
Renal impairment	19 (8.9)	6 (9.5)	202 (23.4)	90 (19.3)	11 (25.0)	47 (22.5)
Anemia	85 (39.9)	19 (30.2)	335 (38.8)	178 (38.2)	17 (38.6)	89 (42.6)
Bone fracture	36 (16.9)	14 (22.2)	182 (21.1)	85 (18.2)	2 (4.5)	26 (12.4)
Pneumonia	19 (8.9)	13 (20.6)	150 (17.4)	62 (13.3)	6 (13.6)	37 (17.7)
**CCI Deyo**						
Mean CCI (SD)	0.7 (1.1)	0.6 (0.9)	1.4 (1.6)	1.4 (1.5)	1.9 (1.6)	1.8 (1.7)
CCI = 0	128 (60.1)	37 (58.7)	339 (39.3)	157 (33.7)	11 (25.0)	67 (32.1)
CCI = 1	47 (22.1)	15 (23.8)	190 (22.0)	139 (29.8)	9 (20.5)	39 (18.7)
CCI = 2	26 (12.2)	9 (14.3)	145 (16.8)	84 (18.0)	8 (18.2)	38 (18.2)
CCI ≥ 3	12 (5.6)	**	189 (21.9)	86 (18.5)	16 (36.4)	65 (31.1)

*ASCT* autologous stem cell transplantation, *CCI* Charlson co-morbidity index, *SD* standard deviation, *Q1/Q3* inter-quartile range, *NA* novel agents (thalidomide or bortezomib) alone, *CCNA* chemotherapy (melphalan cyclophosphamide, vincristine, other chemotherapy regimens) combined with novel agents, *CA* chemotherapy alone, *SA* steroids alone.

^a^From diagnosis date to censor (31 Dec 2017) or death; **To protect patient privacy, all non-zero counts that were less than three were suppressed, including 4 ASCT recipients who received first-line CA or SA.

76% of ASCT recipients diagnosed in the post-bortezomib period received first-line therapy with NA, 22.5% received CCNA, and < 2% received CA or SA. Treatment groups in ASCT recipients did not differ significantly in terms of sex, age, MM co-morbidities or CCI. Among non-transplanted patients, the percentage of adults aged ≥ 65 years treated with NA increased from 14% in the pre-bortezomib period to 49% in the post-bortezomib period, the percentage treated with CCNA changed minimally (29–27%) and use of CA decreased from 25 to 5%, and SA from 31.3 to 19% (Table 1).

### Disease progression models

The DPMs illustrate the percentage of patients who moved through each line of treatment according to the type of first-line therapy received and includes those who died or received ASCT during their disease journey. DPMs for patients diagnosed in the pre-bortezomib period are provided in Figure S1 and S2 by ASCT status.

**Figure 2:**
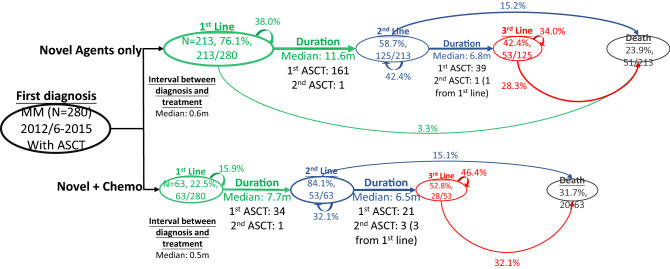
Disease progression model in patients diagnosed with MM in the post-bortezomib periods who received ASCT. The DPMs illustrate the percentage of patients who moved from first-line, to second-line through to the end of third-line treatment according to the type of first-line therapy received and includes those who died or received ASCT during their disease journey. Arrows that return to the line of therapy indicate patients who stayed on the line of therapy for the study duration. Arrows to ‘Death’ indicate the percentage of patients who died at each treatment line. Duration refers to the gap between commencement of one line of therapy and the next. The figures (lengths, widths, areas etc.) are directly proportional to the numbers displayed. *ASCT* autologous stem cell transplant, *MM* multiple myeloma, *Tx* treatment.

Compared to the pre-bortezomib period, in the post-bortezomib period the number of ASCT recipients initiated on NA increased from 41.1% to 76.1%, of whom 36.0% continued first-line treatment until study end, 3.3% died and 58.7% moved to second-line (Fig. 2). The median duration of the first-line treatment before switching to second-line decreased from 17.5 to 11.6 months. Among those who received second-line treatment, 42.4% continued second-line until study end, 15.2% died and 42.4% moved to third-line. The median treatment duration between second and third-line decreased from 14.9 to 6.8 months. The number of patients who died by study end decreased from 46.5 to 23.9%.

The percentage of patients who died increased with each line of treatment in all treatment groups and in both study periods. However, the percentage of all transplanted patients who died by study end decreased from 54.1% to 47.5% between the pre and post-bortezomib periods. This reflects the higher percentage of patients who remained on each line of treatment, the lower percentage who moved to more advanced lines of therapy during the disease journey in the post-bortezomib period, and the shorter follow-up period available in the post-bortezomib period (median 5.69 vs 3.26 years).

In the post-bortezomib period the number of patients with newly diagnosed MM initiated on NA increased from 17.5% in the pre-bortezomib period to 54.6%, of whom 15.1% continued first-line treatment until study end, 33.0% died and 51.9% moved to second-line. The number of patients who died by study end decreased from 74.1 to 60.3%.

Fewer patients were initiated on CA or SA in the post-bortezomib period but the death rate after first-line was unchanged (> 50%). The overall percentage of patients who died decreased in CA recipients from 89.6 to 70.5% in the post-bortezomib period, and in SA recipients from 84.6 to 60.8%.

### Treatment of MM

Treatment line of therapy diagrams for patients who diagnosed in the pre-bortezomib period are provided in Figure S3 and S4 by ASCT status.

**Figure 3:**
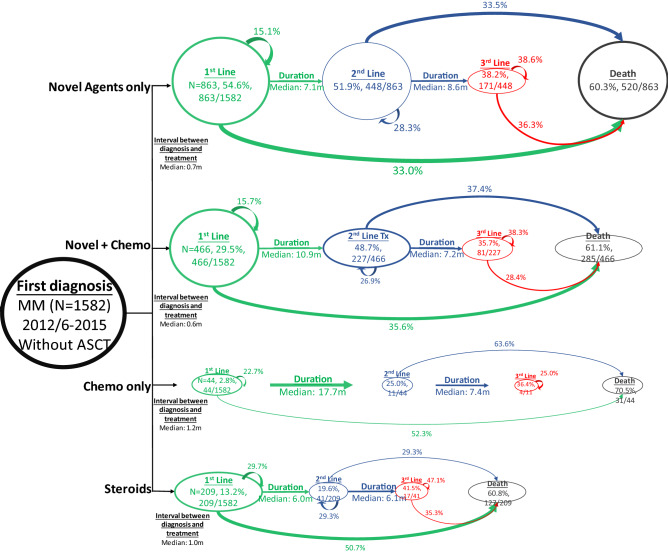
Disease progression model in patients diagnosed with MM in the post-bortezomib period who did not receive ASCT. The DPMs illustrate the percentage of patients who moved from first-line, to second-line through to the end of third-line treatment according to the type of first-line therapy received and includes those who died or received ASCT during their disease journey. Arrows that return to the line of therapy indicate patients who stayed on the line of therapy for the study duration. Arrows to ‘Death’ indicate the percentage of patients who died at each treatment line. Duration refers to the gap between commencement of one line of therapy and the next. The figures (lengths, widths, areas etc.) are directly proportional to the numbers displayed. *ASCT* autologous stem cell transplant, *MM* multiple myeloma, *Tx* treatment.

In ASCT recipients, first-line regimens moved from predominantly thalidomide, chemotherapy and steroids in the pre-bortezomib period, to regimens that included bortezomib in the post-bortezomib period (Figure S3 and Fig. 3). Overall, 98.6% of ASCT recipients diagnosed in the post-bortezomib period received a novel agent during first-line and 90% received bortezomib and 77.5% received combined thalidomide and bortezomib. The percentage of ASCT recipients aged ≥ 65 years who received first-line novel agents increased from 70% to 100% (Table 2).

**Table 2:** First-line treatment regimens in MM patients overall and according to transplantation status by age category.

	Patients who underwent ASCT (N = 526)				Patients who did not receive ASCT (N = 3566)					
	< 65 years		65–74 years		< 65 years		65–74 years		≥ 75 years	
	Pre-bortezomib	Post-bortezomib	Pre-bortezomib	Post-bortezomib	Pre-bortezomib	Post-bortezomib	Pre-bortezomib	Post-bortezomib	Pre-bortezomib	Post-bortezomib
	(n = 236)	(n = 240)	(n = 10)	(n = 39)	(n = 613)	(n = 518)	(n = 661)	(n = 474)	(n = 710)	(n = 590)
**Bortezomib + Thalidomide**	26 (11.0)	178 (74.2)	3 (30.0)	30 (76.9)	50 (8.2)	289 (55.8)	11 (1.7)	191 (40.3)	11 (1.5)	163 (27.6)
Bortezomib + Thalidomide	19 (8.1)	153 (63.8)	3 (30.0)	26 (66.7)	37 (6.0)	257 (49.6)	11 (1.7)	175 (36.9)	11 (1.5)	154 (26.1)
Bortezomib + Thalidomide + chemo	7 (3.0)	25 (10.4)	0	4 (10.3)	13 (2.1)	32 (6.2)	0	16 (3.4)	0	9 (1.5)
**Thalidomide-based**	96 (40.7)	19 (7.9)	*	*	137 (22.3)	57 (11.0)	87 (13.2)	35 (7.4)	103 (14.5)	59 (10.0)
Thalidomide-based	75 (31.8)	11 (4.6)	*	*	112 (18.3)	43 (8.3)	77 (11.6)	27 (5.7)	92 (13.0)	57 (9.7)
Thalidomide-based + Chemo	21 (8.9)	8 (3.3)	0	0 (0.0)	25 (4.1)	14 (2.7)	10 (1.5)	8 (1.7)	11 (1.5)	*
**Bortezomib-based**	4 (1.7)	31 (12.9)	0	4 (10.3)	4 (0.7)	58 (11.2)	3 (0.5)	60 (12.7)	*	91 (15.4)
Bortezomib-based	*	16 (6.7)	0	4 (10.3)	4 (0.7)	36 (6.9)	3 (0.5)	35 (7.4)	*	79 (13.4)
Bortezomib-based + chemo	*	15 (6.3)	0	0 (0.0)	0	22 (4.2)	0	25 (5.3)	0	12 (2.0)
Melphalan + bortezomib + thalidomide	*	6 (2.5)	0	*	3 (0.5)	14 (2.7)	6 (0.9)	32 (6.8)	3 (0.4)	33 (5.6)
Melphalan + thalidomide	14 (5.9)	*	*	*	88 (14.4)	19 (3.7)	188 (28.4)	42 (8.9)	197 (27.7)	55 (9.3)
Melphalan + bortezomib	0	0	*	0	0	12 (2.3)	3 (0.5)	54 (11.4)	3 (0.4)	65 (11.0)
Melphalan-based	8 (3.4)	*	0	0	114 (18.6)	4 (0.8)	183 (27.7)	10 (2.1)	178 (25.1)	13 (2.2)
Cyclophosphamide-based	0	0	0	0	16 (2.6)	4 (0.8)	17 (2.6)	*	12 (1.7)	8 (1.4)
Other mono chemo	8 (3.4)	0	0	0	17 (2.8)	0	8 (1.2)	0	*	0
Two-chemo combination	33 (14.0)	0	3 (30.0)	0	43 (7.0)	3 (0.6)	10 (1.5)	0	3 (0.4)	0
Three-chemo combination	6 (2.5)	0	0	0	*	0	0	0	0	0
Steroid	40 (16.9)	3 (1.3)	0	0	139 (22.7)	58 (11.2)	145 (21.9)	48 (10.1)	197 (27.7)	103 (17.5)

*ASCT* autologous stem cell transplant.

*To protect patient privacy, all non-zero counts that were less than three were suppressed.

In the post-bortezomib period, second-line therapy in ASCT recipients was dominated by lenalidomide (37.6%) which was reimbursed for relapsed refractory MM from December 2012, followed by similar proportions of patients who received bortezomib, cyclophosphamide, or other chemotherapy regimens. Lenalidomide and cyclophosphamide were the most frequently used third-line drugs in both periods.

**Figure 4:**
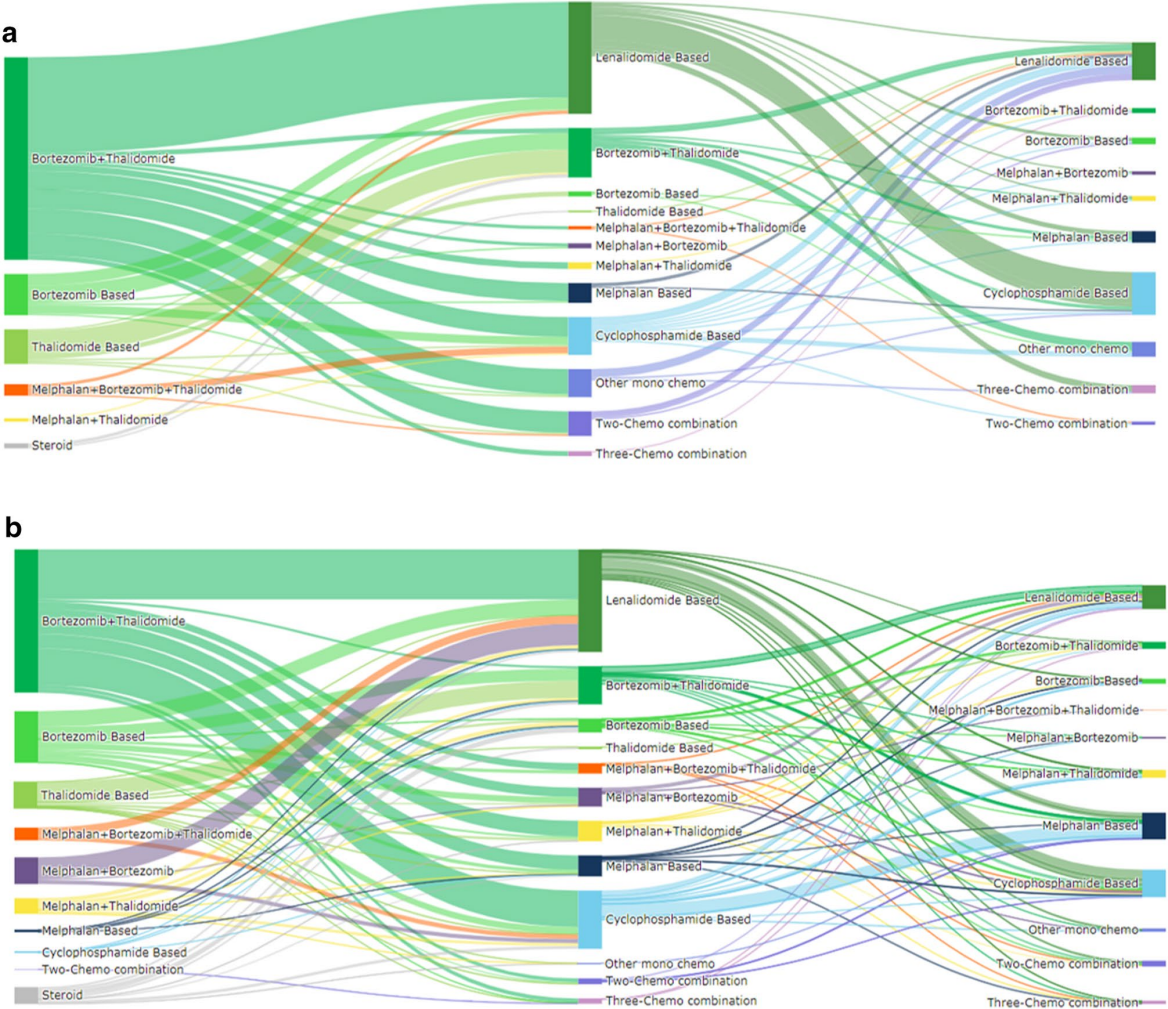
Progression from first to third-line treatment in patients with MM by transplant status in the post-bortezomib period. (**A**) Patients with ASCT. (**B**) Patients without ASCT. *ASCT* autologous stem cell transplantation.

The majority of non-transplanted patients in the pre-bortezomib period received first-line treatment with a regimen that included either thalidomide (44.0%) and/or melphalan (48.0%), or SA (24.2%) (Figure S4), whereas 67.1% received bortezomib in the post-bortezomib period (Fig. 4). The percentage of non-transplanted patients aged ≥ 65 years who received first-line novel agents increased from 45 to 83% (Table 2).

The most significant change in second-line treatments between the pre- and post-bortezomib periods in non-transplanted patients was a marked increase in the use of lenalidomide from 10.1% to 34.4%, and a decrease in the use of melphalan from 32.5% to 23.7%. Third-line treatments in non-transplanted patients showed little change between the pre and post-bortezomib periods.

## Discussion

We evaluated disease progression and lines of therapy in 4092 patients with newly diagnosed and treated MM. By dividing the analysis before and after the date on which bortezomib was recommended for re-imbursement for first-line treatment of MM in Taiwan, we were able to observe the evolution of the MM treatment pathway over time. Unlike studies conducted in individual institutions or studies that use medical records, the NHIRD dataset covers the whole population of Taiwan and reliably captures the entire disease journey from diagnosis. Linking NHIRD data to the death registry minimized the percentage of patients lost to follow-up through death at home or in another institution. Thus, we are confident that we identified all administered treatments and patient deaths regardless of where they occurred.

In contrast to survival curves which provide a static picture of an outcome of interest, DPMs use real-world data to provide a dynamic ‘helicopter’ overview of the entire treatment journey. We constructed DPMs to evaluate progression through lines of treatment and outcomes according to initial treatment, but they can equally be used to evaluate the impacts of new treatments or interventions at different stages of the disease journey, or in specific populations.

In the DPMs we observed marked differences in the characteristics, length and outcomes of the clinical journey in patients who did, and did not, receive ASCT, and between patients initiated on NA compared to other treatment regimens. The DPMs illustrate the relentless progression of MM to relapse and death in most patients, particularly those who are not suitable for ASCT. The benefits of treatment initiation with NA in this population are evident in terms of reduced mortality after each line of treatment and lower overall case fatality during the study period. Nevertheless, there was high percentage of deaths from first- to second-line treatment that increased even further at each subsequent line, even in the post-bortezomib period when most patients received two novel agents for first-line. Our results highlight the ongoing unmet medical needs in the frontline setting of MM treatment. In view of the high attrition rate between first and second-line, novel agents with new modes of action should be used at the earliest line possible. Intervals between first and second-line treatment were shorter than 12 months for most treatment types, and intervals progressively shortened with each treatment line^12^.

Compared to the pre-bortezomib period, the post-bortezomib period was characterized by a substantially higher percentage of patients who remained on a line of therapy until study end, a lower percentage who died by study end, lower rates of progression to more advanced treatment lines, but little change in death rates during each line of therapy. This apparent discrepancy is probably due to the older age of MM patients, including ASCT recipients in the post-bortezomib period, reflecting a propensity to approach MM treatment in elderly patients more aggressively. New onset of MM in older age is associated with significantly higher International Staging System scores than younger patients, which are based on parameters not measured in our study, such as beta-2-microglobulin and albumin levels^13^. High levels of beta-2-microglobulin are associated with frailty and renal impairment^14^, and may underly the poorer outcome of older patients, despite use of novel therapies^13^. It is worth noting that a population-based study using SEER data in the US showed no improvement in 10-year relative survival rates among adults aged ≥ 75 years from 1993 until 2012^15^, highlighting the continued unmet medical need for effective treatments in this age group.

Re-imbursement of bortezomib for first-line treatment, together with evidence of increased efficacy when bortezomib is combined with another novel agent^5^, has led to combined thalidomide and bortezomib becoming the most common first-line treatment regimen in the post-bortezomib era, irrespective of transplant status, and contributes to 50% of all first-line MM treatments in Taiwan. Consequently, melphalan and cyclophosphamide are now more commonly used in salvage settings. Lenalidomide comprised 31.5% of all second and third-line treatments in the post-bortezomib period versus 15.5% in the pre-bortezomib period. Our post-bortezomib period observations are similar to other countries such as Europe and the US after availability of novel agents, where 48% of first-line treatments included bortezomib, and up to 86% of patients received novel agents for first-line treatment^1,16,17^.

There are 17 qualified transplantation centers in Taiwan, and more than 90% of transplants are conducted in 8 major centers. All ASCT in MM is performed in the inpatient setting. The overall rate of ASCT increased modestly over the study period and was 15.4% in the post-bortezomib period. This is close to the rate of 19.8% reported across seven countries in Asia^18^, and of 16.2% in the US between 2006–2014 in a large cohort of patients enrolled in a health insurance database^8^, but contrasts with 37.8% among non-Hispanic whites estimated from a US transplant registry in 2013, and 23–56% of MM patients in Europe^16,19^. Among patients aged < 65 year at diagnosis, 28% received ASCT. The percentage of older patients with MM who received ASCT increased from 0.7 to 3.6% but remained low, reflecting the fear of increased transplant-related morbidity and mortality in older adults in Taiwan. Increasing evidence supports that novel agents and ASCT improve survival in older adults without excess toxicity, although these findings have yet to translate into firm recommendations for the use of ASCT in seniors^3,20–22^. ASCT is used in patients with MM up to 70 years of age in Europe and 75 years in the US^21^. In the US, 52% of all patients who received ASCT for MM between 2008 and 2013 were aged 61–75 years^19^.

The vast majority of non-transplanted patients were aged ≥ 65 years and the percentage who received a novel agent as part of their initiation regimen approximately doubled, to a majority (83%) in the post-bortezomib period. However, fewer older patients received combined thalidomide and bortezomib, and there was more use of melphalan-based regimens as patients aged at the time of diagnosis. The higher CCI score in patients who received first-line treatment with SA suggests poorer fitness for more advanced therapy in these patients. However, the CCI score was the same across the other three exposure groups (NA, CCBA and CA) which suggests that other factors may influence drug choice in older patients.

Potential limitations of this observational study include lack of clinical information on disease stage, disease progression and response to therapy, which did not allow us to directly link treatment with clinical benefit. We used progression through lines of treatment and death as indicators of disease progression. The duration of follow-up was shorter in the post-bortezomib versus the pre-bortezomib periods, which may have influenced our results. The NHIRD does not identify patients participating in clinical trials, receiving drugs through the Compassionate Use Program in Taiwan, or patients who are self-paying, usually for third-line or salvage treatments. However, these numbers are likely to be low.

In conclusion, the treatment landscape of MM in Taiwan has evolved. Despite the increased use of combined bortezomib and thalidomide for first-line and of lenalidomide for second and/or third-line, mortality rates among patients with MM remain high. Age remains a key determinant of treatment choice, rates of relapse are high, and MM remains incurable. Many patients with MM die before receiving second-line treatment and novel agents with different modes of action should be used as early as possible during treatment to maximize their benefits. There is an ongoing unmet need for additional treatment options in patients with MM. DPMs provide a granular view of the disease journey, can be used for evaluating the effectiveness of new interventions and can contribute to therapeutic decision making.

## Methods

Taiwan’s National Health Insurance is a single-payer system in place since 1995^23^, that provides mandatory health insurance for the entire population of Taiwan. The system provides comprehensive population coverage and accessibility to all health services. All administrative and claims data are held centrally in the NHIRD-population-based claims database provided by the Taiwan National Health Insurance Administration and maintained by the Health and Welfare Data Science Center, Ministry of Health and Welfare, Executive Yuan, Taiwan^23^. Primary and secondary diagnoses are coded in the International Classification of Diseases, Ninth Revision, Clinical Modification (ICD-9-CM) format. Information relating to patient demography and the type and date of all health services provided is recorded and verified by the National Health Insurance Administration^24–26^.

All patients who were newly diagnosed with MM (ICD-9 codes 203.0 × or 203.0 or 203) from 01 January 2007 to 31 December 2015 were captured from the NHIRD. The cohort analysis included all patients aged ≥ 18 years at diagnosis, who had been in the database at least for 12 months prior to the MM diagnosis index date, who had at least one hospital or outpatient visit for MM after the diagnosis date, and who had received treatment for their MM. Patients with a record of any other primary cancer before the diagnosis date were excluded. Patients with any diagnosis of plasma cell leukaemia (203.1 ×) or other immunoproliferative neoplasms (203.8 ×) within 2 months of the first MM diagnosis indicating rapid disease progression, were also excluded.

A line of therapy was considered to be ended when there was at least a 60-day gap in any treatment included in that line of therapy; or the addition of a new treatment to the existing regimen after > 90 days; or death, or the end of the data. New treatments added within 90 days after starting a line of therapy were considered an addition to the existing line.

Patients were followed up until death, end of the follow-up period (31 December 2017), or dis-enrolment from the database, whichever occurred first. Co-morbidities present 12 months prior to the diagnosis were recorded and the Charlson Comorbidity Index (CCI) calculated using ICD-9-CM (Charlson/Deyo). For MM-associated comorbidities, patients were required to have had ≥ 3 outpatient visit claims or ≥ 1 hospitalization claim with a primary or secondary diagnosis of anemia (ICD-9 281, 283, 284, 285, 776), renal injury/renal failure (586), pneumonia (486), or bone fracture (800–829). Deaths were captured from the Taiwan Death Registry linked to the NHIRD.

The use of chemotherapy (cyclophosphamide, melphalan, bendamustine, vincristine, etoposide, liposomal doxorubicin, cisplatin), novel agents (bortezomib, thalidomide, lenalidomide) and steroids for first and subsequent lines of treatment of MM given as an outpatient or inpatient was coded using Reimbursement Codes defined by the National Health Insurance Administration. Drugs were captured by Anatomical Therapeutic Chemical codes. Treatments patterns were assessed by identifying first-, second- and third-lines of therapy from prescription start and end dates.

Additional information about the NHIRD, inclusion and exclusion criteria for the cohort analysis and the definition of a line of therapy, are provided in the supplement.

All personally identifiable information was encrypted to protect patients. The study was granted an exemption from ethical review by the Taipei Medical University-Joint Institutional Review Board and an exemption from the need for patient consent. The study was conducted according to all applicable guidelines and regulations.

### Statistical analysis

DPMs were constructed using a Markov model with results displayed using Sankey diagrams. The Markov approach models the probabilities of different treatment states and the rates of transitions among them, based on the dependencies of current information with previous information. We used Markov models to predict the probability of different disease states, including transition from one line of treatment to the next and death. Sankey diagrams with horizontal and vertical nodes were generated to visualize treatment transition from first to third-line in patients who did/did not receive treatment. Analyses in each period were stratified by ASCT status. All analyses were performed using SAS Version 9.4 (Cary, NC, USA).

## Supplementary Information

Supplementary Information.
