# Supplementary material for: Treatment pathways and disease journeys differ before and after introduction of novel agents in newly diagnosed multiple myeloma in Taiwan
Source: Sci Rep. 2021 Jan 13;11:1112. doi: 10.1038/s41598-020-80607-4 (PMC7806818; doi:10.1038/s41598-020-80607-4)

Treatment pathways and disease journeys differ before and after introduction of novel agents in newly diagnosed multiple myeloma in Taiwan

Yanfang Liu, Chao-Hsiun Tang, Hong Qiu, Sara Siggins, and Hsin-An Hou

Supplement

Table S1 Demographic, clinical characteristics and follow-up duration of patients with treated MM diagnosed in the pre-bortezomib period (01 Jan 2007 until 31 May 2012) by ASCT status and first-line treatment

	Patients who underwent ASCT (n=246)				Patients without ASCT (n=1984)			
	NA	CCNA	CA	SA	NA	CCNA	CA	SA
Variables	N=101	N=47	N=58	N=40	N=348	N=550	N=605	N=481
**Sex n (%)**								
Male	61 (60.4)	24 (51.1)	39 (67.2)	20 (50.0)	186 (53.4)	333 (60.5)	356 (58.8)	261 (54.3)
Female	40 (39.6)	23 (48.9)	19 (32.8)	20 (40.0)	162 (46.6)	217 (39.5)	249 (41.2)	220 (45.7)
**Age (mean, SD)**	54.4 (6.9)	55.3 (6.5)	52.3 (8.4)	53.3 (6.6)	(67.1,12.4)	(70.9,10.4)	(68.7,11)	(70.2,12.6)
18-29	0 (0.0)	0 (0.0)	0 (0.0)	0 (0.0)	0 (0.0)	0 (0.0)	0 (0.0)	0 (0.0)
30-39	**	0 (0.0)	5 (8.6)	**	6 (1.7)	7 (1.3)	4 (0.7)	3 (0.6)
40-49	25 (24.8)	9 (19.1)	13 (22.4)	10 (25.0)	14 (4.0)	11 (2.0)	29 (4.8)	27 (5.6)
50-59	47 (46.5)	22 (46.8)	31 (53.4)	20 (50.0)	82 (23.6)	48 (8.7)	98 (16.2)	65 (13.5)
60-69	27 (26.7)	15 (31.9)	8 (13.8)	8 (20.0)	92 (26.4)	157 (28.5)	166 (27.4)	97 (20.2)
70-79	0 (0.0)	**	**	0 (0.0)	98 (28.2)	212 (38.5)	208 (34.4)	165 (34.3)
≧80	0 (0.0)	0 (0.0)	0 (0.0)	0 (0.0)	56 (16.1)	115 (20.9)	100 (16.5)	122 (25.4)
**Follow up duration* (years)**								
Mean (SD)	5.63 (1.9)	5.47 (1.8)	5.52 (3.2)	6.33 (2.9)	3.28 (2.8)	3.06 (2.5)	2.88 (2.8)	3.08 (3.1)

Med (range)	6.05 (0.95-9.42)	6.15 (1.44-8.17)	4.86 (0.61-10.91)	6.96 (1.30-10.41)	2.22 (0.04-10.42)	2.2 (0.05-9.43)	1.84 (0.02-10.99)	1.73 (0.02-10.77)
Q1, Q3	4.47, 6.88	4.52, 6.62	2.82, 8.65	3.53, 8.85	0.65, 6.08	0.83, 5.36	0.74, 4.13	0.47, 5.62
**Comorbidity n (%)**								
Renal impairment	8 (7.9)	5 (10.6)	6 (10.3)	4 (10.0)	67 (19.3)	93 (16.9)	81 (13.4)	85 (17.7)
Anemia	31 (30.7)	15 (31.9)	10 (17.2)	10 (25.0)	129 (37.1)	207 (37.6)	217 (35.9)	195 (40.5)
Bone fracture	24 (23.8)	9 (19.1)	5 (8.6)	4 (10.0)	57 (16.4)	114 (20.7)	107 (17.7)	76 (15.8)
Pneumonia	7 (6.9)	4 (8.5)	5 (8.6)	**	53 (15.2)	83 (15.1)	83 (13.7)	97 (20.2)
**CCI Deyo (mean, SD)**	0.6 (0.9)	0.8 (1.3)	0.5 (0.8)	0.7 (1.0)	1.3 (1.5)	1.2 (1.4)	1.2 (1.4)	1.4 (1.5)
CCI=0	62 (61.4)	28 (59.6)	38 (65.5)	23 (57.5)	137 (39.4)	217 (39.5)	253 (41.8)	172 (35.8)
CCI=1	22 (21.8)	11 (23.4)	12 (20.7)	9 (22.5)	86 (24.7)	158 (28.7)	140 (23.1)	114 (23.7)
CCI=2	9 (8.9)	5 (10.6)	6 (10.3)	5 (12.6)	70 (20.1)	76 (13.8)	109 (18.0)	92 (19.1)
CCI≧3	8 (7.9)	3 (6.4)	**	3 (7.5)	55 (15.8)	99 (18.0)	103 (17.0)	103 (21.4)

CCI, Charlson co-morbidity index; SD, standard deviation; Q1/Q3 inter-quartile range; NA, novel agents (thalidomide or bortezomib) alone; CCNA, chemotherapy (melphalan cyclophosphamide, vincristine, other chemotherapy regimens) combined with novel agents; CA, chemotherapy alone; SA, steroids alone.

*From diagnosis date to censor (31 Dec 2017) or death; ** To protect patient privacy, all non-zero counts that were less than three were suppressed

Figure S1 Disease Progression Models in patients diagnosed with MM in the pre-bortezomib period who received ASCT

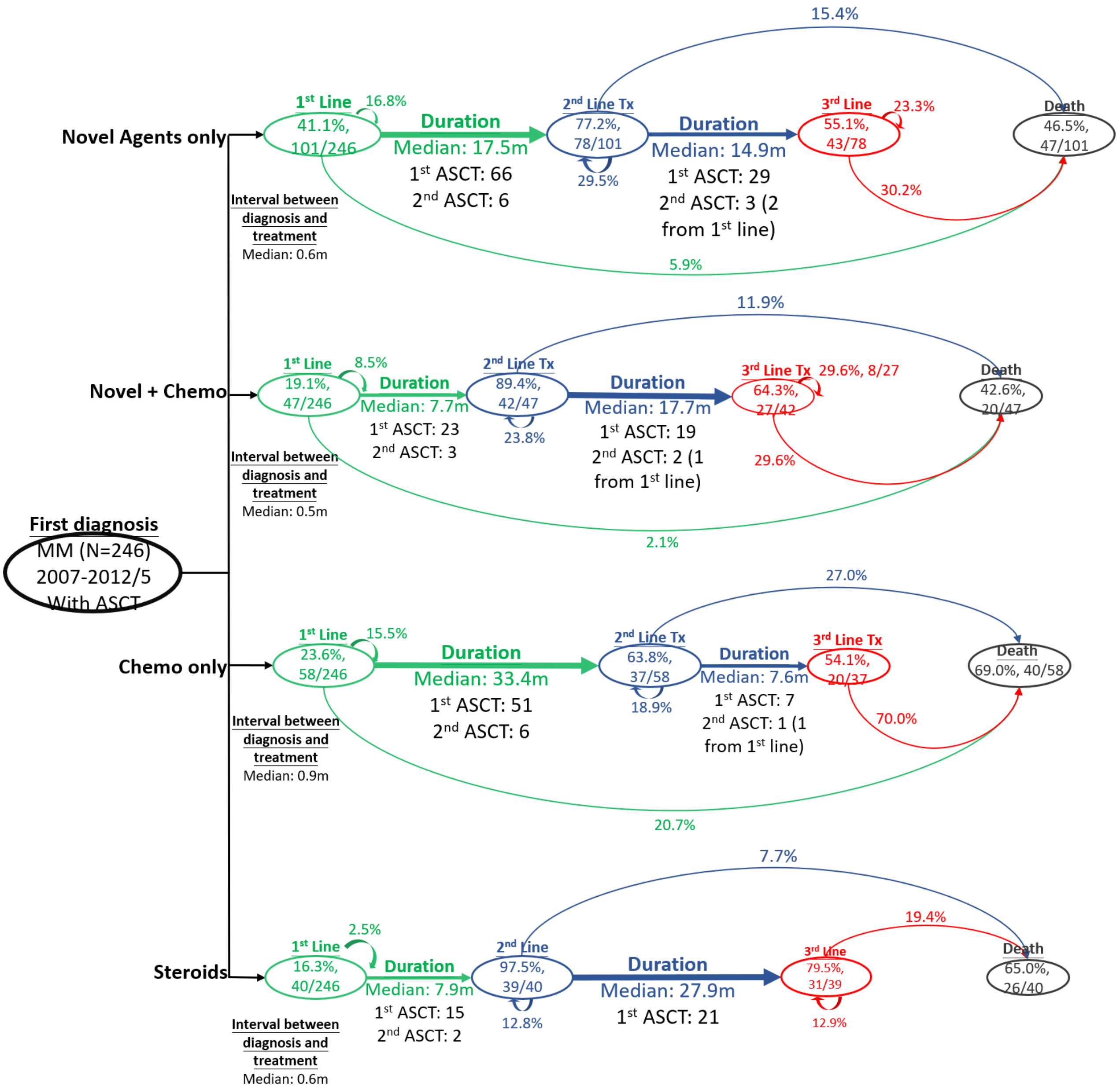

The DPMs illustrate the percentage of patients who moved from first-line, to second line through to the end of third-line treatment according to the type of first line therapy received and includes those who died or received ASCT during their disease journey. Arrows that return to the line of therapy indicate patients who stayed on the line of therapy for the study duration. Arrows to 'Death' indicate the percentage of patients who died at each treatment line. Duration refers to the gap between commencement of one line of therapy and the next. The figures (lengths, widths, areas etc) are directly proportional to the numbers displayed.

ASCT, autologous stem cell transplant; MM, multiple myeloma; Tx, treatment

Figure S2 Disease Progression Models in patients diagnosed with MM in the pre-bortezomib period who did not receive ASCT

The DPMs illustrate the percentage of patients who moved from first-line, to second line through to the end of third-line treatment according to the type of first line therapy received and includes those who died or received ASCT during their disease journey. Arrows that return to the line of therapy indicate patients who stayed on the line of therapy for the study duration. Arrows to 'Death' indicate the percentage of patients who died at each treatment line. Duration refers to the gap between commencement of one line of therapy and the next. The figures (lengths, widths, areas etc) are directly proportional to the numbers displayed.

ASCT, autologous stem cell transplant; MM, multiple myeloma; Tx, treatment

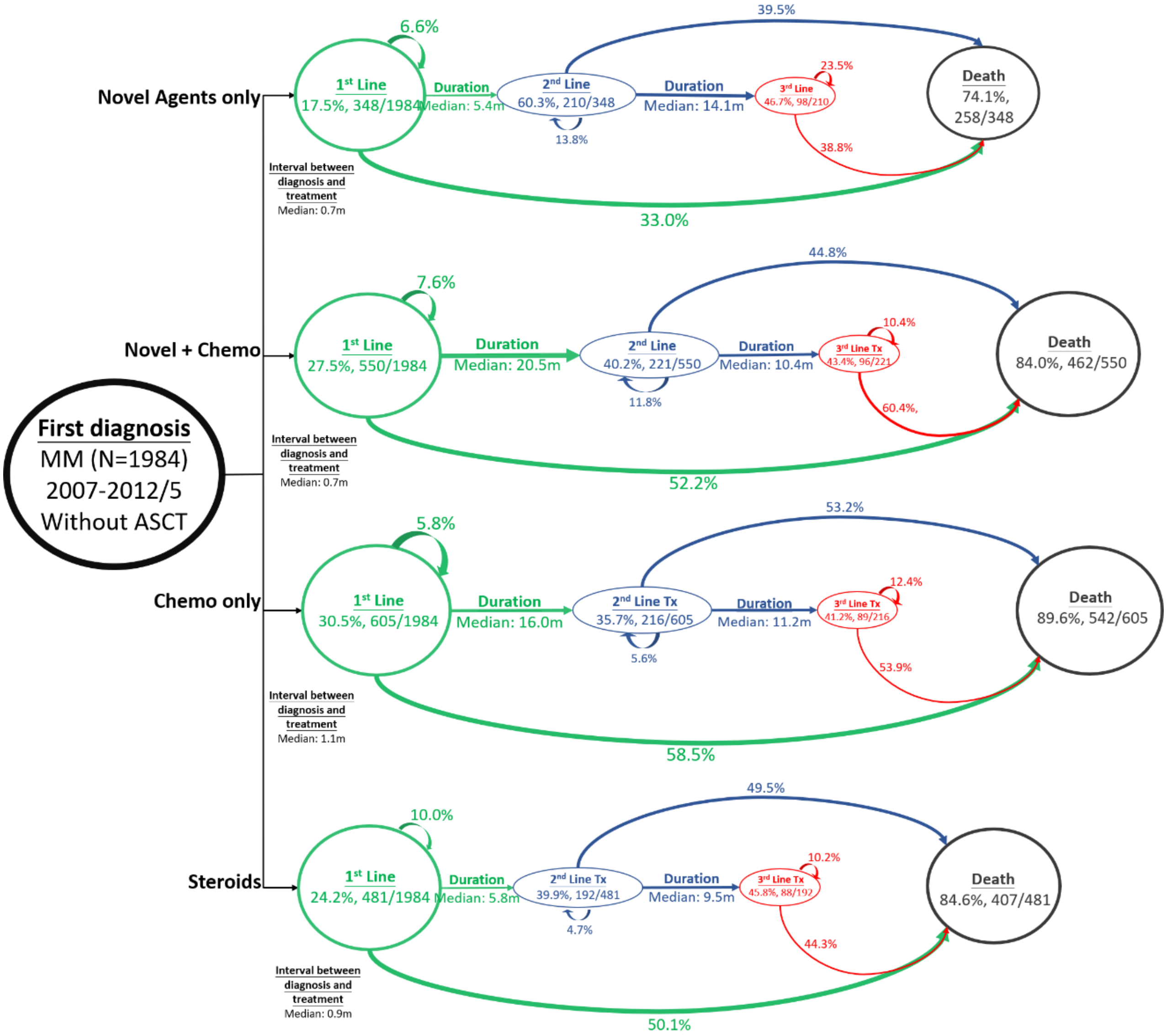

Figure S3 Progression from first to third-line treatment in patients with MM diagnosed in the pre-bortezomib period who received ASCT

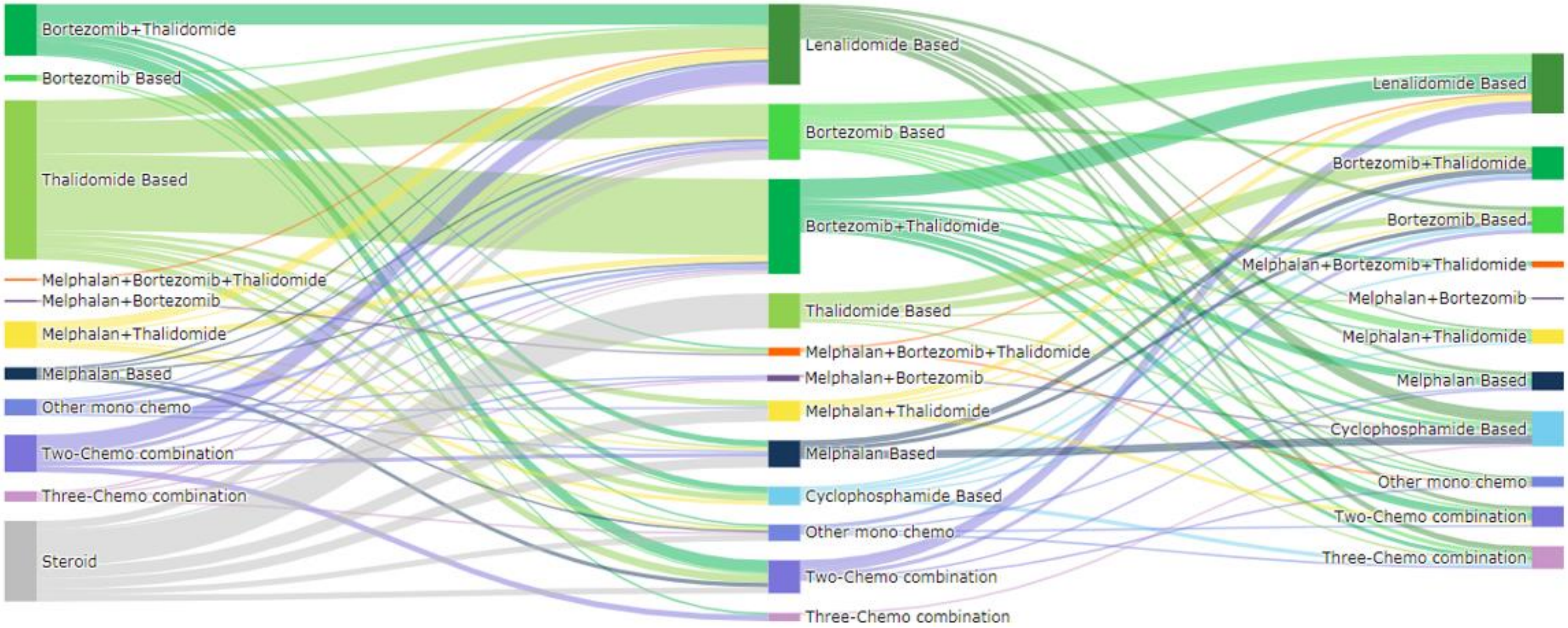

Figure S4 Progression from first to third-line treatment in patients with MM diagnosed in the pre-bortezomib period who did not receive ASCT

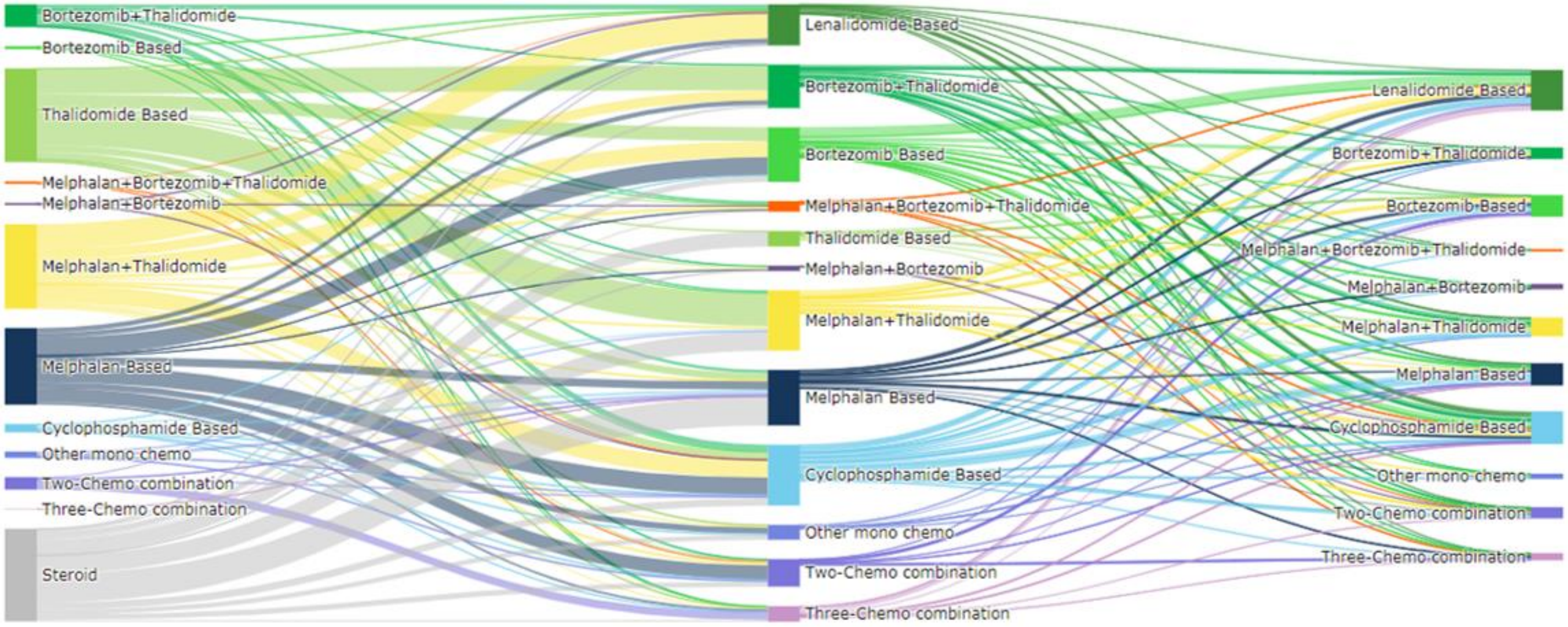

Supplement: Supplementary file 1 — Supplementary Information. [file 41598_2020_80607_MOESM1_ESM.pdf]
